# Mutation I136V alters electrophysiological properties of the Na_V_1.7 channel in a family with onset of erythromelalgia in the second decade

**DOI:** 10.1186/1744-8069-4-1

**Published:** 2008-01-02

**Authors:** Xiaoyang Cheng, Sulayman D Dib-Hajj, Lynda Tyrrell, Stephen G Waxman

**Affiliations:** 1 Department of Neurology, Yale University School of Medicine, New Haven, Connecticut 06510, USA; 2 Center for Neuroscience and Regeneration Research, Yale University School of Medicine, New Haven, Connecticut 06510, USA; 3 Rehabilitation Research Center, Veterans Affairs Connecticut Healthcare System, West Haven, Connecticut 06516, USA

## Abstract

**Background:**

Primary erythromelalgia is an autosomal dominant pain disorder characterized by burning pain and skin redness in the extremities, with onset of symptoms during the first decade in the families whose mutations have been physiologically studied to date. Several mutations of voltage-gated Na^+ ^channel Na_V_1.7 have been linked with primary erythromelalgia. Recently, a new substitution Na_V_1.7/I136V has been reported in a Taiwanese family, in which pain appeared at later ages (9–22 years, with onset at 17 years of age or later in 5 of 7 family members), with relatively slow progression (8–10 years) to involvement of the hands. The proband reported onset of symptoms first in his feet at the age of 11, which then progressed to his hands at the age of 19. The new mutation is located in transmembrane segment 1 (S1) of domain I (DI) in contrast to all Na_V_1.7 mutations reported to date, which have been localized in the voltage sensor S4, the linker joining segments S4 and S5 or pore-lining segments S5 and S6 in DI, II and III.

**Results:**

In this study, we characterized the gating and kinetic properties of I136V mutant channels in HEK293 cells using whole-cell patch clamp. I136V shifts the voltage-dependence of activation by -5.7 mV, a smaller shift in activation than the other erythromelalgia mutations that have been characterized. I136V also decreases the deactivation rate, and generates larger ramp currents.

**Conclusion:**

The I136V substitution in Na_V_1.7 alters channel gating and kinetic properties. Each of these changes may contribute to increased excitability of nociceptive dorsal root ganglion neurons, which underlies pain in erythromelalgia. The smaller shift in voltage-dependence of activation of Na_V_1.7, compared to the other reported cases of inherited erythromelalgia, may contribute to the later age of onset and slower progression of the symptoms reported in association with this mutation.

## Background

Voltage-gated Na^+ ^channels are heteromeric protein complexes, consisting of a pore-forming α subunit and auxiliary β subunits [1]. Nine α subunits (Na_V_1.1–Na_V_1.9) have been reported to date, and have been shown to be expressed in a tissue-specific manner [2]. Each α subunit is composed of four homologous domains (DI-DIV), with each domain consisting of six transmembrane segments (S1–S6), with S4 acting as a voltage-sensor and S5 and S6 lining the pore [1]. Voltage-gated Na^+ ^channels are essential for the production of action potentials and are thus pivotal for excitability of neurons, myocytes, and neuroendocrine cells [2]. Mutations of voltage-gated Na^+ ^channels have been linked to a number of human diseases including epilepsy, periodic paralysis, cardiac disorders, and pain disorders [3-7].

Primary erythromelalgia is a rare, inherited, autosomal dominant disorder, characterized by intermittent burning pain in the extremities and skin redness of the affected areas. Episodes can be triggered by warm stimuli or mild exercise [8]. The onset of symptoms varies from childhood, adolescence, to adulthood [9], with onset of pain most commonly occurring during the first decade of life. Primary erythromelalgia has been linked to mutations in *SCN9A*, the gene that encodes voltage-gated sodium channel Na_V_1.7 [10]. Na_V_1.7 is preferentially expressed in nociceptive dorsal root ganglia (DRG) neurons and sympathetic ganglia neurons [11-14], and it produces a fast-activating and -inactivating TTX-sensitive (TTX-S) current with a slow recovery from inactivation [12,15]. Na_V_1.7 also exhibits slow kinetics of closed-state inactivation, which enables it to respond to small, slow depolarizing inputs and boost subthreshold depolarizations [15]. Thus, gain-of-function mutations of the Na_V_1.7 channel might be expected to contribute to the hyperexcitability of nociceptive neurons which underlies chronic pain in patients with erythromelagia.

Nine mutations of Na_V_1.7 have been identified in patients with primary erythromelalgia, with eight of them, all from families with onset in childhood (≤ 10 years of age), having been characterized by electrophysiological studies [16-22]. All eight mutations cause a hyperpolarizing shift in voltage dependence of activation in Na_V_1.7 channels. Slow deactivation kinetics and larger ramp currents were observed in all but the F1449V mutant channels [16-22]. The changes in the biophysical properties of mutant Na_V_1.7 are predicted to increase excitability of DRG neurons and thereby contribute to the pathophysiology of erythromelalgia. In fact, three of these mutations (F1449V, A863P, and L858H) have been studied using current clamp and shown to increase excitability of DRG neurons in culture [18,20,23].

Recently, a new erythromelalgia mutation of Na_V_1.7 was reported by Lee et al in a Taiwanese family, affecting family members of three generations [24], with a later age of onset. In this family, pain appeared first in the feet at ages varying from 9 to 22 years, with onset at ages ≥ 17 years in five of seven family members; pain was reported in the hands in one-half of the affected family members, 8–10 years later (C-C Yang and M-J Lee, personal communication). The clinical phenotype was unusual in that the proband reported onset of pain in feet at 11 years of age, later than in most of familial cases [16,18,21,22,25] and in the patients with *de novo *founder mutations that have been studied to date [19,20]. Genetic analysis identified a substitution of isoleucine 136 with valine (I136V) in Na_V_1.7 in all affected family members, but not in unaffected family members or control DNA [24]. This amino acid substitution is located in DIS1 of the Na_V_1.7 channel, in contrast to all Na_V_1.7 mutations reported to date, which have been localized in the voltage sensor S4, the linker joining segments S4 and S5 or pore-lining segments S5 and S6 in DI, II and III. Here, we investigate the effect of this mutation on biophysical properties of Na_V_1.7 channels.

## Results

### I136V mutation produces lower threshold for activation, slower kinetics of fast inactivation, and larger window currents

**Figure 1:**
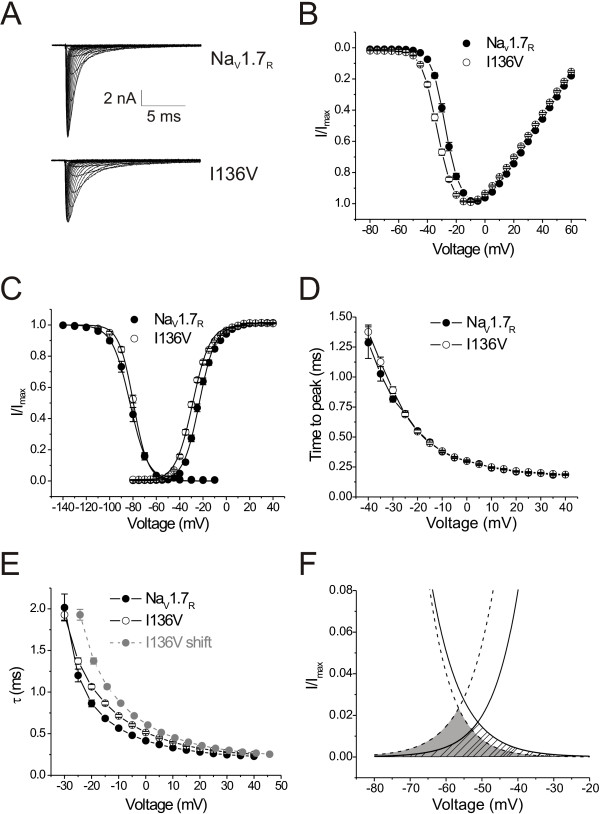
**I136V mutation alters voltage-dependent activation and steady-state fast inactivation of Na_V_1.7 channels**. **A**, Representative family traces of Na+ currents from voltage-clamped HEK293 cells expressing either wild-type Na_V_1.7_R _(top) or I136V mutant (bottom) channels. Cells were held at -100 mV, and Na^+ ^currents were elicited by step depolarizations from -80 to +60 mV in 5 mV increment every 5 seconds. **B**, Normalized peak current-voltage relationship for Na_V_1.7_R _(n = 40) and I136V mutant (n = 43) channels. **C**, Comparison of the voltage-dependent activation and steady-state fast inactivation of Na_V_1.7_R _and I136V mutant channels. A hyperpolarizing shift (-5.7 mV) of activation was observed in I136V mutant channels. Steady-state fast inactivation was examined using a series of 500-ms prepulses from -140 mV to -10 mV followed by 40-ms test pulses at -10 mV. I136V mutation did not change the V_1/2,fast _but altered the slope factor. **D**, Activation kinetics, measured as time-to-peak, were similar between Na_V_1.7_R _and I136V mutant channels. **E**, Fast inactivation kinetics of Na_V_1.7_R _and I136V mutant channels. Inactivation time constants were calculated by fitting the decay phases of currents shown in Figure 1A with single-exponential function. Gray circles represent I136V shifted 5.7 mV in a depolarizing direction to match the voltage-dependent activation of wild type Na_V_1.7_R _channels. The inactivation kinetics of I136V channels are slower than that of Na_V_1.7_R _channels. **F**, Expanded view of overlap of activation and inactivation Boltzmann fits (area as predicted window current) from **C **(shaded area for I136V mutation, and lined area for Na_V_1.7_R_).

Sodium currents were recorded from HEK293 cells stably expressing either wild type Na_V_1.7_R _or I136V mutant channels. Figure 1A shows representative families of inward Na^+ ^current traces of wild type and I136V mutant channels. Cell capacitance was similar (p > 0.05) in cells that were transfected with Na_V_1.7_R _or with I136V mutant channels (Na_V_1.7_R_: 19.2 ± 0.6 pF, n = 40; I136V: 18.9 ± 0.5 pF, n = 43). Figure 1B shows the mean current-voltage relationship of wild-type and I136V channels, with no apparent difference of reversal potentials (Na_V_1.7_R_: 71.5 ± 0.5 mV; I136V: 70.3 ± 0.4 mV). However, currents generated by I136V mutant channels activated and reached the maximal amplitudes at more hyperpolarized potentials compared with wild type channels. Boltzmann fits of conductance show that the I136V mutation significantly shifted the midpoint of the activation curve in a hyperpolarizing direction (I136V: V_1/2,act _= -28.8 ± 0.5 mV, n = 43; Na_V_1.7_R_: -23.1 ± 0.7 mV, n = 40, p < 0.05, Figure 1C, Table 1).

**Table 1:** Electrophysiological properties of wild-type and I136V mutant Na_V_1.7 channels.

Channel	Activation			Fast Inactivation			Slow Inactivation				Ramp Current		
	
	V_1/2,act_	Slope	n	V_1/2,inact_	Slope	n	V_1/2,inact_	Slope	R_in_	n	% of I_max_	V_peak_	n
Na_V_1.7_R_	23.1 ± 0.7	6.40 ± 0.14	40	82.5 ± 1.3	6.94 ± 0.15	15	-66.0 ± 1.6	13.6 ± 0.3	0.19 ± 0.02	11	0.23 ± 0.02	-42.3 ± 0.9	19
I136V	28.8 ± 0.5*	7.13 ± 0.10*	43	79.7 ± 0.6	6.00 ± 0.27*	13	-74.0 ± 0.9*	8.7 ± 0.2*	0.11 ± 0.01*	11	0.79 ± 0.04*	-48.0 ± 0.5*	26

*p < 0.05, I136V vs Na_V_1.7_R_.

Activation kinetics, measured as time-to-peak, were similar between wild-type and I136V channels (Figure 1D). The kinetics of fast inactivation, which reflects the transition from the open to inactive state, were measured by fitting the decaying phase of Na^+ ^currents in Figure 1A with single exponential functions. Because open state inactivation is coupled to activation state [26], the curve of inactivation kinetics of I136V mutant channels was shifted by 5.7 mV in a depolarizing direction (gray circles in Figure 1E) to match the similar activation voltages of wild type channels. Analysis of the data shows that the I136V substitution leads to slower kinetics of fast inactivation.

Steady-state fast inactivation was tested with a series of 500-ms prepulses from -140 to -10 mV followed by a 20 ms test pulse at -10 mV. Although mutant channels showed less inactivation at -100 and -90 mV, no difference was observed in V_1/2,fast inact _between the two channels, probably because the inactivation curve of mutant channels is steeper than that of wild type channels (Figure 1C, Table 1). Figure 1F shows the predicted window current which is defined by the overlap of activation and steady-state fast inactivation curves. I136V mutant channels are predicted to produce larger window currents than wild type channels, and thus are expected to lead to a more depolarized resting membrane potential in DRG neurons [20,23].

### I136V mutant channels exhibit slower deactivation and enhanced steady-state slow inactivation

**Figure 2:**
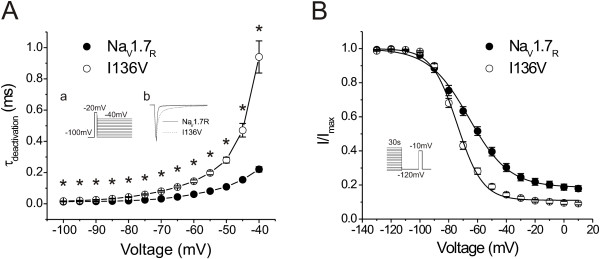
**I136V mutation alters deactivation and steady-state slow-inactivation**. **A**, Time constants of channel deactivation. Cells were held at -100 mV and tail currents were generated by a brief 0.5-ms depolarization to -20 mV followed by a series of repolarizations ranging from -100 to -40 mV to elicit tail currents (insert a). I136 mutant channels (n = 19) show significantly slower deactivation than Na_V_1.7_R _channels (n = 17) at all testing potentials. Insert b shows the representative tail currents from two HEK293 cells expressing Na_V_1.7_R _(solid line) or I136V mutant channels (dotted line). **B**, I136V mutation significantly enhances steady-state slow-inactivation. Steady-state slow-inactivation was examined by a series of prepulses (30 s) from -130 to +10 mV followed by 100-ms return pulse to -120 mV, then a 20-ms test pulse to -10 mV.

Similar to most Na_V_1.7 mutations that cause primary erythromelalgia, I136V mutation significantly decreases the deactivation rate of sodium currents at all tested deactivation potentials (Figure 2A). At -40 mV, the time constant of deactivation was 0.22 ± 0.02 ms (n = 17) for wild type channels and 0.94 ± 0.10 ms (n = 19) for I136V mutant channels. Slower deactivation has been suggested to contribute to neuronal excitability by prolonging responses after the cessation of a depolarizing stimulus [17].

Steady-state slow inactivation was enhanced in mutant channels (Figure 2B). The V_1/2,slow inact _was shifted by 8 mV in a hyperpolarizing direction, and the slope factor was significantly reduced when compared with wild type Na_V_1.7_R _channels. The fraction of I136V channels that are resistant to slow inactivation was reduced compared to wild type channels (Table 1).

### I136V mutant channels recover faster from fast inactivation at -70 mV and generate larger ramp currents, compared to wild type channels

**Figure 3:**
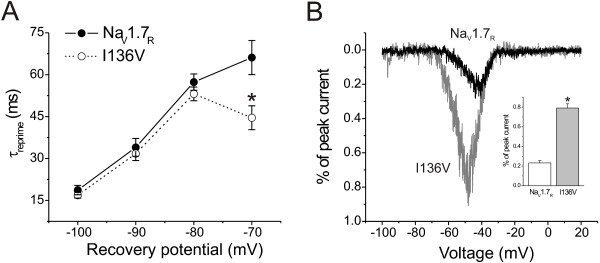
**I136V mutation alters recovery from fast-inactivation and increases currents elicited by slow ramp depolarizations**. **A**, Cells were held at -100 mV, and fast-inactivation was initiated by a 20-ms depolarization to 0 mV, followed by a recovery period (2–300 ms) at a recovery potential, followed by a 10-ms test pulse to 0 mV to measure the available channels. Recovery rate were calculated by comparing the peak currents of test pulse to prepulse at 0 mV (20 ms) after various recovery durations (2–300 ms) at different recovery potentials (-100, -90, -80, and -70 mV), and plotted as a function of recovery potentials. Recovery time constants of Na_V_1.7_R _and I136V mutant channels were then estimated using single-exponential fits. At a recovery potential of -70 mV, I136V mutant channels (n = 11) recovered faster than wild type Na_V_1.7_R _channels (n = 12). **B**, Representative ramp currents from Na_V_1.7_R _(black) and I136V mutant (gray) channels. HEK293 cells were held at -100 mV and a depolarizing voltage ramp from -100 mV to +20 mV was applied at a rate of 0.2 mV/ms. The insert shows mean ramp currents of wild type Na_V_1.7_R _and I136V mutant channels. Currents were normalized to the maximal peak currents from step depolarizations in Figure 1B. I136V mutant channels were activated at more negative potentials and generated larger ramp currents than Na_V_1.7_R _channels (Na_V_1.7_R_: 0.23 ± 0.02, n = 19; I136V: 0.79 ± 0.04, n = 26, p < 0.05).

The ability of channels to recover from fast inactivation (repriming) may determine how fast a neuron can repetitively fire. Repriming kinetics of wild type and mutant channels were examined for a series of recovery periods at four different recovery potentials between prepulses and test pulses. I136V mutation does not change the repriming kinetics at -100, -90, and -80 mV, but significantly enhances the recovery at -70 mV (Figure 3A).

The responses of wild type and mutant channels to slow ramp depolarizations were also tested. HEK293 cells expressing mutant channels produced larger ramp currents than cells expressing wild type channels (I136V: 0.79 ± 0.04%, n = 26; WT: 0.23 ± 0.02%, n = 19, p < 0.05). The I136V mutation also shifted the onset of the currents to more negative potentials (I136V: -48.0 ± 0.5 mV, n = 26; WT: -42.3 ± 0.9 mV, n = 19, p < 0.05).

## Discussion

In this study we investigated the gating properties of the I136V mutation of Na_V_17 channels, which has been linked to inherited primary erythromelalgia in a Taiwanese family reported by Lee et al [24]. Compared to wild-type channels, the I136V substitution leads to a hyperpolarizing shift of activation, slower deactivation kinetics, and larger ramp currents. The I136V mutation did not alter voltage-dependent steady-state fast-inactivation, but decreased the slope factor. The significant changes in the slopes of the activation and steady-state inactivation Boltzman fits might be due to altered allosteric interactions among the four voltage sensor domains. Mutant channels also reprimed faster than wild-type channels but only at a recovery voltage of -70 mV. These changes of the biophysical properties of Na_V_1.7 are expected to contribute to hyperexcitability of DRG neurons, which underlies painful symptoms of erythromelalgia.

I136V mutant channels significantly enhanced slow-inactivation which would be expected to reduce excitability of DRG neurons. However, enhanced slow inactivation has been reported in other erythromelalgia mutations, suggesting that enhanced voltage-dependence of Na_V_1.7 activation dominates over slow inactivation in these erythromelalgia mutations [17,21]. Computer simulations of the effect of mutant Na_V_1.7 channels in model DRG neurons have in fact shown that the hyperpolarized shift in the activation of channels is the key factor in inducing neuron hyperexcitability, whereas slow inactivation modulates the effect on the firing frequency of these neurons [22].

The location (DIS1) and conservative nature of the I136V substitution might account for the milder effect of this mutation on the channel gating properties. Among the eight erythromelalgia mutations characterized so far, four are located within the S4–S5 linkers of DI [21] or DII [17,19], one in DIS4 [16], one each in DIS6 [22] and DIIIS6 [18], and one in DIIS5 [20]. Considering the importance of these regions for channel function [1], it is not surprising that those mutations alter Na_V_1.7 activity. In contrast, I136V is located within DIS1, a segment which has not been shown to have a dominant effect in channel gating. Moreover, both Val and Ile are neutral, nonpolar branched amino acids which differ by a single methyl group in their side chain, and substitution of one residue by the other is generally not expected to cause significant change in protein function. However, a similarly conservative substitution of a serine with threonine (S241T) in the DI S4–S5 linker [27] has been proven to have a significant effect on channel activation, an effect which is likely caused by the larger side chain of the threonine residue compared to that of serine [21]. Thus, the I136V substitution might influence the packing of the S1 segment within the voltage sensor domain (S1–S4) [28] and this could lead to altered gating properties by favoring the open state of the channel.

The conservation of an isoleucine in DIS1 of most voltage-gated sodium and calcium channels is consistent with a role of this residue in channel function. A recent study identified a conserved motif (F-XXX-I-XXX-**I**-XX-N/S-XXX-M/L; the residue in boldface corresponds to I136 in Na_V_1.7) within S1 segments of most voltage-gated Na^+ ^and Ca^2+ ^channels [29]. However, Na_V_1.9 is an exception because the residue which corresponds to I136 in DIS1 is valine [30-32], similar to the mutant Na_V_1.7 from the Taiwanese family [24]. Na_V_1.9 activates at more hyperpolarized potentials compared to the other sodium channels [31,33], and while the S1 sequence of Na_V_1.9 carries additional amino acid changes compared to the other channels, it is possible that the equivalent residue of Na_V_1.7/I136 within Na_V_1.9 contributes to the hyperpolarized activation of this channel. Interestingly, M145T in DIS1 in Na_V_1.1 (which corresponds to the last conserved residue in the S1 consensus motif) has been linked to familial febrile seizures and the activation curve of the mutant Na_V_1.1 has been reported to shift by 10 mV in a depolarizing direction [34]. The opposite effects on voltage-dependence of channel activation of I136V and M145T could reflect a fundamental difference in the structure of the voltage sensor domain (S1–S4) as a consequence of conservative (I136V) and non-conservative (M145T) substitutions of key residues in S1.

The mutation I136V causes a smallest shift (-5.7 mV) in voltage-dependent activation compared to the other erythromelalgia mutations that have been studied to date [16-22]. The small shift induced by I136V is possibly related to the location of the mutation within DIS1 and the conservative nature of the substitution, but despite the reduced magnitude of the shift in I136V, it appears to be enough to contribute to the erythromelalgia symptoms. It is possible that the smaller shift in channel activation is a factor that contributes to the later onset and relatively slower progression of the symptoms from feet to hands in this family. This case may thus represent a correlation of genotype and phenotype in patients with primary erythromelalgia.

Recent studies have revealed an association between missense mutations within other parts of Na_V_1.7 and another painful neuropathy, paroxysmal extreme pain disorder (PEPD) [35], whereas nonsense mutations of Na_V_1.7 have been linked to channelopathy-associated congenital indifference to pain [36-38]. The identification and characterization of mutant Na_V_1.7 channels have begun to elucidate the molecular basis for positive symptoms (pain) and negative symptoms (indifference to pain) in patients carrying these mutations. The present results document another gain-of-function mutation of Na_V_1.7 that is associated with painful symptoms in humans, in a family with onset of erythromelalgia's symptoms in the second decade, and add to the data pointing to this channel as a target for therapeutic intervention for the treatment of chronic pain.

## Conclusion

I136V mutation in DIS1 of Na_V_1.7 channels has been reported to cause primary erythromelalgia in a Taiwanese family, with onset of symptoms in the second decade. Electrophysiological study revealed a hyperpolarizing shift of the voltage-dependence of activation, decreased deactivation rate, and a larger response to slow ramp depolarization in mutant channels. All these changes could contribute to the development of primary erythromelalgia. The shift in activation is smaller when compared with other primary erythromelalgia mutations which present with pain at earlier ages, possibly due to the conservative nature of the substitution between isoleucine and valine and the location of this mutation in the DIS1 in Na_V_1.7. This study adds to the evidence for an essential role of Na_V_1.7 in the pain sensory pathway, and suggests that the extent of shift of voltage-dependence of activation of Na_V_1.7 may be a factor that is correlated with the rate of development of erythromelalgia symptoms.

## Methods

### Plasmids and stable cell lines

The TTX-resistant human Na_V_1.7_R _expression vector has been described previously [39]. The I136V substitution was introduced into Na_V_1.7_R _using the QuickChange XL site-directed mutagenesis kit (Stratagene, La Jolla, CA). Human embryonic kidney cells (HEK293) stably expressing either wild-type (Na_V_1.7_R_) or mutant (I136V) channels were generated as described before [21].

### Electrophysiology

Whole-cell voltage-clamp recordings of HEK293 cells expressing either wild type Na_V_1.7_R _or I136V mutant channels were performed at room temperature (20–22°C) using an Axopatch 200 B amplifier (Axon Instruments, Foster City, CA). Fire-polished electrodes (0.6–1.3 MΩ) were fabricated from 1.6 mm outer diameter borosilicate glass micropipettes (World Precision Instruments, Sarasota, FL). The pipette potential was adjusted to zero before seal formation, and liquid junction potential was not corrected. Capacity transients were cancelled and voltage errors were minimized with 80–90% series resistance compensation. Currents were acquired with Clampex 9.2, 5 min after establishing whole-cell configuration, sampled at a rate of 50 or 100 kHz, and filtered at 10 kHz.

For current-voltage relationships, cells were held at -100 mV and stepped to a range of potentials (-80 to +60 mV in 5 mV increments) for 50 ms. Peak inward currents (I) were plotted as a function of depolarization potential to generate I-V curves. Activation curves were obtained by converting I to conductance (G) at each voltage (V) using the equation *G *= *I*/(*V-V*_*rev*_), where V_rev _is the reversal potential which was determined for each cell individually. Activation curves were then fit with Boltzmann function in the form of *G *= *G*_*max*_/{1 + *exp*[(*V*_1/2,*act*_-*V*)/*k*]}, where *G*_max _is the maximal sodium conductance, *V*_1/2,*act *_is the potential at which activation is half-maximal, *V *is the test potential, and *k *is the slope factor.

Steady-state fast inactivation was achieved with a series of 500-ms prepulses (-140 to 10 mV in 10 mV increments) and the remaining non-inactivated channels were activated by a 20 ms step depolarization to -10 mV. Steady-state slow inactivation was determined with 30 s prepulses ranging from -130 to +10 mV followed by a 100 ms hyperpolarization at -120 mV to remove fast inactivation. Remaining available channels were activated by a 20 ms test pulse to -10 mV. Peak inward currents obtained from steady-state fast inactivation and slow inactivation protocols were normalized with the maximal peak current (I_max_) and fit with Boltzman functions:

*I*/*I*_*max *_= 1/{1 + *exp*[(*V *- *V*_1/2,*inact*_)/*k*]}

for fast inactivation, and

*I*/*I*_*max *_= *R*_*in *_+ (1 - *R*_*in*_)/{1 + *exp*[(*V *- *V*_1/2,*inact*_)/*k*]}

for slow inactivation, where

*V *represents the inactivating prepulse potential, *V*_1/2,*inact *_represents the midpoint of inactivation curve, and *R*_*in *_is the fraction of channels that are resistant to inactivation.

Deactivation was examined using a short (0.5 ms) depolarizing pulse to -20 mV followed by a 50 ms repolarizing pulse to potentials ranging from -100 to -40 mV with 5 mV increments. Deactivation kinetics was calculated by fitting the decaying currents with a single exponential function. Ramp currents were elicited with a slow depolarizing voltage ramp from -100 to +20 mV at a rate of 0.2 mV/ms. The absolute ramp current amplitude was normalized to the maximal peak current obtained by I-V protocol.

The pipette solution contained (in mM): 140 CsF, 10 NaCl, 1 EGTA, 10 dextrose, and 10 HEPES, pH 7.35 (adjusted with CsOH), and the osmolarity was adjusted to 307 mOsmol/L with sucrose. The extracellular bath solution contained (in mM): 140 NaCl, 3 KCl, 1 MgCl_2_, 1 CaCl_2_, 10 dextrose, 10 HEPES, pH 7.4 (adjusted with NaOH), and the osmolarity was adjusted to 311 mOsmol/L with sucrose. Tetrodotoxin (TTX, 300 nM) was added to the bath solution to block endogenous voltage-gated sodium currents in HEK293 cells, permitting currents from wild type Na_V_1.7_R _or I136V to be recorded in isolation.

### Data Analysis

Data were analyzed using Clampfit 9.2 (Molecular Devices) and Origin 7.5 (Microcal Software, Northampton, MA), and presented as means ± SE. Statistical significance was determined by unpaired student's *t *tests.

## Competing interests

The author(s) declare that they have no competing interests.

## Authors' contributions

XC collected, analyzed and interpreted electrophysiological data. SDD-H participated in the experimental design and interpretation of the data. LT generated the mutant I136V construct and established the stable cell lines. SGW conceived the project, participated in the experimental design and interpretation. XC, SDD-H, and SGW participated in writing of the manuscript. All authors read and approved the final manuscript.
